# Molecular Engineering Enhances the Charge Carriers Transport in Wide Band-Gap Polymer Donors Based Polymer Solar Cells

**DOI:** 10.3390/molecules25184101

**Published:** 2020-09-08

**Authors:** Siyang Liu, Shuwang Yi, Peiling Qing, Weijun Li, Bin Gu, Zhicai He, Bin Zhang

**Affiliations:** 1 School of Materials Science and Engineering, Baise University, Baise 533000, China; ray-lsy@hotmail.com (S.L.); plqing110@163.com (P.Q.); liweijun_bsu@163.com (W.L.); 18276637865@163.com (B.G.); 2 Institute of Polymer Optoelectronic Materials and Devices, State Key Laboratory of Luminescent Materials and Devices, South China University of Technology, Guangzhou 510640, China; shuwangyi399@sina.com (S.Y.); zhicaihe@scut.edu.cn (Z.H.); 3 Jiangsu Engineering Laboratory of Light-Electricity-Heat Energy-Converting Materials and Applications, School of Materials Science and Engineering, Changzhou University, Changzhou 213164, China

**Keywords:** molecular engineering, polymer donors, high hole mobility, polymer solar cells

## Abstract

The novel and appropriate molecular design for polymer donors are playing an important role in realizing high-efficiency and high stable polymer solar cells (PSCs). In this work, four conjugated polymers (PIDT-O, PIDTT-O, PIDT-S and PIDTT-S) with indacenodithiophene (IDT) and indacenodithieno [3,2-b]thiophene (IDTT) as the donor units, and alkoxy-substituted benzoxadiazole and benzothiadiazole derivatives as the acceptor units have been designed and synthesized. Taking advantages of the molecular engineering on polymer backbones, these four polymers showed differently photophysical and photovoltaic properties. They exhibited wide optical bandgaps of 1.88, 1.87, 1.89 and 1.91 eV and quite impressive hole mobilities of 6.01 × 10^−4^, 7.72 × 10^−4^, 1.83 × 10^−3^, and 1.29 × 10^−3^ cm^2^ V^−1^ s^−1^ for PIDT-O, PIDTT-O, PIDT-S and PIDTT-S, respectively. Through the photovoltaic test via using PIDT-O, PIDTT-O, PIDT-S and PIDTT-S as donor materials and [6,6]-phenyl-C-71-butyric acid methyl ester (PC_71_BM) as acceptor materials, all the PSCs presented the high open circuit voltages (*V_oc_s*) over 0.85 V, whereas the PIDT-S and PIDTT-S based devices showed higher power conversion efficiencies (PCEs) of 5.09% and 4.43%, respectively. Interestingly, the solvent vapor annealing (SVA) treatment on active layers could improve the fill factors (FFs) extensively for these four polymers. For PIDT-S and PIDTT-S, the SVA process improved the FFs exceeding 71%, and ultimately the PCEs were increased to 6.05%, and 6.12%, respectively. Therefore, this kind of wide band-gap polymers are potentially candidates as efficient electron-donating materials for constructing high-performance PSCs.

## 1. Introduction

The unique advantages of polymer solar cells (PSCs), such as mechanical flexibility, semi-transparency and large-scale production, mean they are the most promising next-generation photovoltaic technologies in the future [1]. Recently, the highest power conversion efficiency (PCE of > 18% has been achieved, which is potentially possible for commercial application [2].

In order to optimize the generation of electricity in PSCs, various methods are used in PSCs, including the development of new active materials and device engineering. Generally, bulk-heterojunction (BHJ) structure is a widely utilized device technique, which comprises at least two types of semiconducting components in photoactive layer, where the electron donors (D) support the electrons, and electron acceptors (A) transport electrons. To obtain high-performance PSCs, the formation of interpenetrating networks between donor and acceptor domains, within nano-scale sizes and the connectivity among these two phases, deposit the significant influences on the charge carrier transport, recombination and collection in BHJ solar cell [3,4,5]. Furthermore, an electron donor is usually a conjugated polymer, while the acceptor is often a fullerene derivatives, small molecules and polymers. Among these materials, they take part in light absorption, exciton generation and exciton dissociation. Furthermore, they also play a role in transporting charge carriers through respective electrodes to the external circuit. In this regard, the development on the donors and acceptors are essential in adjusting the optical, electrical, and photovoltaic properties in PSCs.

At present, one of the important acceptors, used in PSCs, are fullerene derivatives, such as [6,6]-phenyl-C-61-butyric acid methyl ester (PC_61_BM), [6,6]-phenyl-C-71-butyric acid methyl ester (PC_71_BM), and 1′,1″,4′,4″-tetrahydro-di [1,4] methanonaphthaleno [5,6] fullerene-C60 (ICBA) [6]. Fullerenes and their derivatives exhibit ultrafast electron transfer from conjugated polymers by their versatile isotropic electron transport properties. This is due to their large conjugated spheres and electron-deficient centre. This characteristic can facilitate charge separation by delocalizing charges, low internal reorganization energy for electron transfer. The large spherical size of fullerenes increases tolerance to disorder and elimination of disturbances by the presence of donor polymer. In addition, it helps electrons find effective tunnel out of the mixed phase regions, and the entropy effects decrease the Coulomb barrier for charge separation [7,8]. Overall, fullerene and its derivatives are useful electron-accepting materials in achieving high-efficiency PSCs.

With the development of polymer donors, the PSCs combine a wide band-gap polymer donor and a narrow band-gap fullerene acceptor, and have great potential in achieving relatively good performance [9]. The design strategy for polymer donors that match with small-molecule acceptors requires: (1) Suitable energy levels, which could be finely adjusted by introducing heteroatoms or functional groups, and offering a channel for charge carriers transport among the small-molecule acceptors [10]; (2) the side chain or backbone modification provides moderate solubility, together with proper hole mobility, aggregation properties and prior molecular orientation for the optimal phase separation [11,12,13]; and (3) a broad and complementary absorption spectrum for harvesting much more sunlight. Hence, the proper design on molecular structure would realize the high-performance polymer donors for PSCs.

Molecular conformation has proven that polymer backbone planarity could affect energy levels, BHJ morphology, energetic disorder and charge transport dramatically. To control the molecular structure, noncovalent intramolecular interactions that favor a certain intramolecular conformation could be the suitable way for molecular structure design. In particular, sulphur-oxygen interaction is known to increase conjugated backbone planarity in a D-A copolymer, leading to high charge carrier mobility and device performance [14]. Instead of simply decreasing rotatable single bonds between units, the use of these conformational strategies may provide excellent control over organic semiconducting properties, and allow for more facile approaches to controlling molecular structure. For instance, Kim et al. introduced the ortho-hydrogen to promote a more planar conjugated backbone by sulphur-oxygen interactions, leading to the improvement on morphology and degradation [15]. Ma and co-workers optimized two BHJ polymers’ structures to realize the high charge carrier mobility, long lifetime, and great free-carrier diffusion length [16]. Zhang et al. also achieved a high PCE of 9.0% using a conjugated polymer as donor, with a non-fullerene ITIC as the acceptor, with a remarkably low energy loss of 0.53 V, but without any negative impacts on the morphology of the blend films [17]. Liang et al. summarized that the silicon, germanium, sulfur and nitrogen as bridge atoms can change the degree of coplanarity between consecutive backbone units, and more effectively, by flattening the π-conjugated molecular framework to tailor the physical properties of IDT based p-type materials [18]. Currently, PSCs are focused on materials synthesis and device engineering, where both of these efforts are dedicated to further improve the photovoltaic performance. However, few investigations have been performed to explore the fundamental properties of polymer materials, which is extremely important and helpful in understanding the relationship between materials and device performance, as well as potentially providing guidelines to design novel materials and device architecture.

In this work, we designed and synthesized four planar D-A copolymers (PIDT-O, PIDTT-O, PIDT-S, PIDTT-S) based on the indacenodithiophene (IDT), indacenodithieno [3,2-b]thiophene (IDTT), alkoxy-substituted benzoxadiazole and benzothiadiazole derivatives [19,20,21,22]. These four polymers displayed wide band-gap properties with optical bandgap around 1.9 eV. Through the photovoltaic characterization via using these polymers as donors and PC_71_BM as acceptor, it was found that the alkoxy-substituted benzothiadiazole based polymers (PIDT-S and PIDTT-S) showed higher hole mobilities than the alkoxy-substituted benzoxadiazole based polymers (PIDT-O and PIDTT-O). Under the association of solvent annealing in device engineering, both of PIDT-S and PIDTT-S gave the final PCEs exceeding 6.0%, while the other two polymers of PIDT-O and PIDTT-O showed lower PCEs around 4%. We found that such an imbalance between photovoltaic performance and charge carrier transport properties can be attributed toward the hole mobilities and blend film morphologies. Therefore, it is believed that these results will be beneficial for understanding the charge transport-morphology-performance relationship for efficient and stable PSCs.

## 2. Results and Discussion

### 2.1. Synthesis

**Scheme 1:**
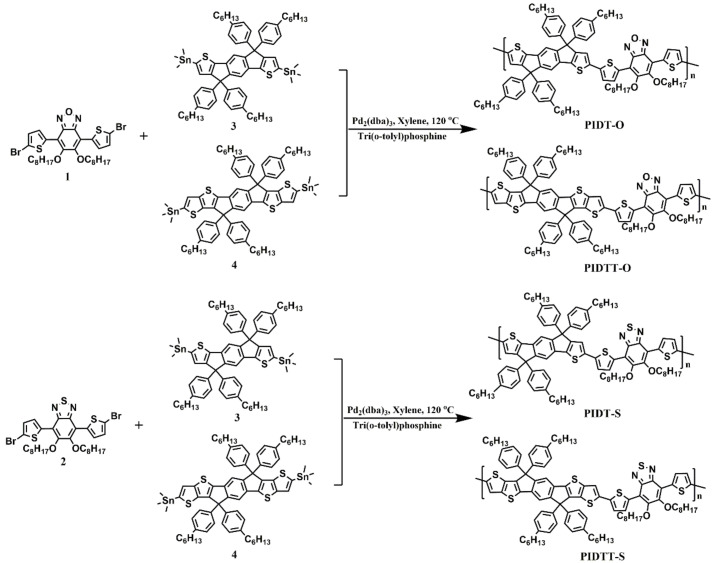
Synthetic routes of the polymers.

To understand the synthesis distinctly, the detailed synthetic routes of PIDT-O, PIDTT-O, PIDT-S and PIDTT-S are shown in Scheme 1. All polymers were synthesized by the general Stille polycondensation reaction in the presence of active Pd_2_(dba)_3_ as the catalyst and tri-o-tolylphopine as the ligand. All reactions were performed in 120 °C, and then the crude polymers were purified via the Soxhlet extraction through the methanol, acetone, hexane and chloroform, respectively. Finally, the final chloroform solution from Soxhlet extraction was precipitated in dry methanol again to attain the target polymers. All of the polymers displayed the red solid, and showed high solubility in the general organic solvents, such as dichloromethane, chloroform, tetrahydrofuran, toluene and chlorobenzene. In order to get the molecular weights of PIDT-O, PIDTT-O, PIDT-S and PIDTT-S, we used the Gel Permeation Chromatography (GPC) characterization with tetrahydrofuran as the eluent and polystyrene as the internal standards to test number-averaged molecular weights (*M_n_s*) and weight-averaged molecular weights (*M_w_s*). It was found that the *M_n_s* and *M_w_s* were 26,300, 43,700, 26,800, 49,400 and 45,000, 91,200, 53,400, 120,100, with the polydispersity index (PDIs) of 1.71, 2.09, 1.99 and 2.34 for PIDT-O, PIDTT-O, PIDT-S and PIDTT-S, respectively. (Table 1) From the test of molecular weights, we can see that all of the polymers show very high molecular weights, which are significantly beneficial for the solution-processible technique in PSCs.

### 2.2. Thermal Properties

**Figure 1:**
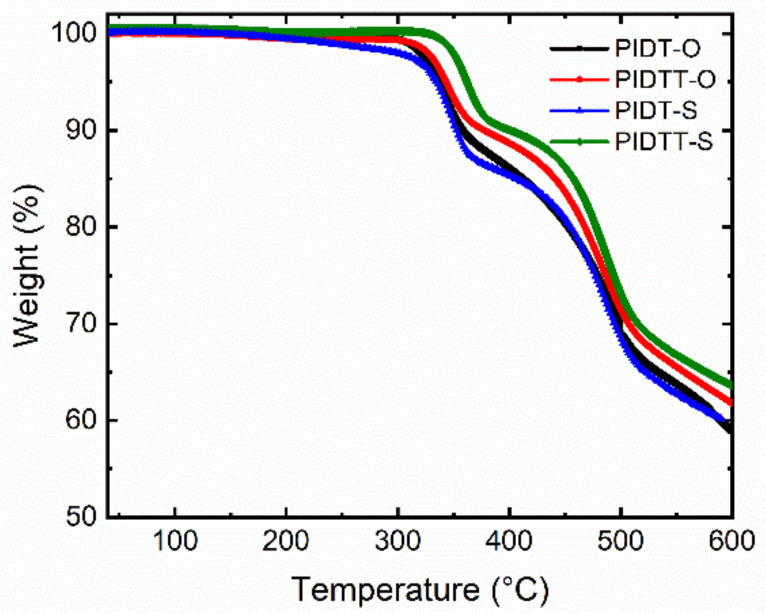
Thermal gravimetric analysis curves of PIDT-O, PIDTT-O, PIDT-S and PIDTT-S.

To evaluate thermal stability, the thermal gravimetric analysis (TGA) under N_2_ was used to study the thermal properties of PIDT-O, PIDTT-O, PIDT-S and PIDTT-S, and the related TG curves and data were presented in Figure 1 and Table 1, respectively. As shown in Figure 1, the degradation temperature (*T_d_*) at 5% weight loss for PIDT-O, PIDTT-O, PIDT-S and PIDTT-S are 337, 343, 335, and 362 °C, respectively. It is noted that all of these four polymers display the very high thermal stability, which is potentially beneficial for the PSCs.

### 2.3. UV-vis Absorption and Electrochemical Properties

**Figure 2:**
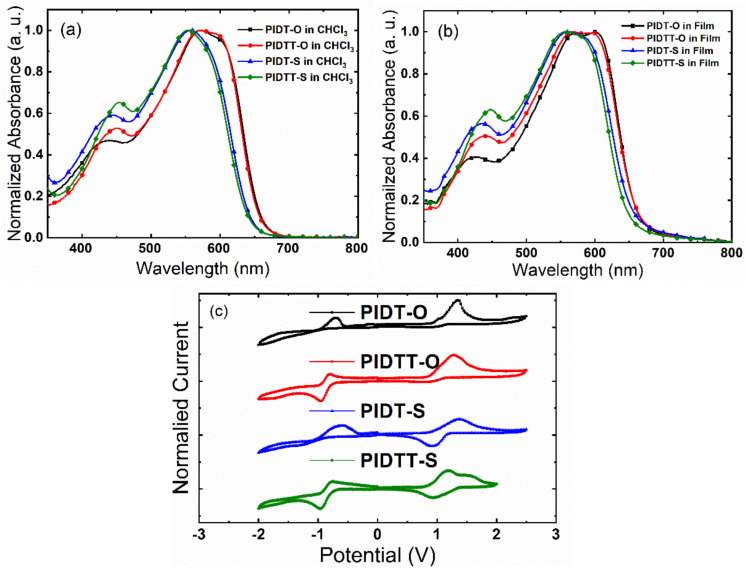
(**a**) UV-visible spectra in chloroform and (**b**) in film, (**c**) Cyclic voltammograms curves of PIDT-O, PIDTT-O, PIDT-S and PIDTT-S.

The UV-vis absorption spectra of PIDT-O, PIDTT-O, PIDT-S and PIDTT-S in chloroform and in solid film were shown in Figure 2a,b, and the corresponding data were summarized in Table 2. All of them display the similar absorption spectra with two major absorption bands. The absorption spectra in chloroform shows two peaks at 439 and 573 nm for PIDT-O, 453 and 574 nm for PIDTT-O, 447 and 556 nm for PIDT-S, and 453 and 553 nm for PIDTT-S, respectively. The absorption peaks in short wavelength (<450 nm) are assigned to the π-π* transition of the conjugated rigid polymers, whereas the absorption peaks in the range of 530–610 nm are attributed to the intramolecular charge transfer (ICT) transition [23]. In the film state, all the polymers show almost the same features of absorption as that in solution, except PIDT-O and PIDTT-O have one more shoulder peak at 600 and 598 nm, respectively. From the Figure 2b, the absorption spectra in the film state also show two distinct peaks ranged from 350 to 700 nm. Compared with the absorption in the solution, the absorption in the solid state gives the obvious red shift. This may be attributed to the stronger intermolecular interactions between the planar π-conjugated skeletons [24]. Accordingly, this interaction between conjugated backbones could influence solubility and miscibility of bulk-heterojunction blends in solution state, but also affect π-π stacking and crystallization in solid-state films. Finally, the PIDT-O, PIDTT-O, PIDT-S and PIDTT-S show the absorption onsets of 660, 661, 655 and 650 nm with the optical band gaps of 1.88, 1.87, 1.89, and 1.91 eV, respectively, which indicate that they are wide band-gap polymers.

The electrochemical properties of PIDT-O, PIDTT-O, PIDT-S and PIDTT-S were characterized by cyclic voltammetry (CV) in dry acetonitrile. The oxiditative/reductive potentials (*E_ox_* and *E_red_*) were calibrated from CV curves with the ferrocene/ferrocenium (*Fc*/*Fc*^+^) as the internal standard and the tetra-n-butyl ammonium hexafluorophosphate (TBAF, 0.1 M) as the supporting electrolyte. The corresponding CV curves were shown in Figure 2c and the detailed data were summarized in Table 2. From Figure 2c, it displays the obviously reversible oxidation curves with *E_ox_s* of 0.92 V for PIDT-O, 0.84 V for PIDTT-O, 0.87 V for PIDT-S, and 0.85 V for PIDTT-S, respectively. Furthermore, the reversible reduction curves with *E_red_s* of −0.85 V for PIDT-O, −0.79 V for PIDTT-O, −0.89 V for PIDT-S, and −0.78 V for PIDTT-S, respectively, are also recorded. The highest occupied molecular orbital (HOMO) and the lowest unoccupied molecular orbital (LUMO) energy levels were calculated according to the empirical formula of *E_HOMO_* = −*e*(*E_ox_* + 4.8 − *E*_1/2, (*Fc/Fc*_^+^_)_) and *E_LUMO_* = −*e*(*E_red_* + 4.8 − *E*_1/2, (*Fc/Fc*_^+^_)_), where the *E*_1/2, (*Fc/Fc*+)_ was recorded as 0.38 V. Therefore, the HOMOs of PIDT-O, PIDTT-O, PIDT-S and PIDTT-S are −5.34, −5.26, −5.29 and −5.25 eV and the LUMOs are −3.57, −3.63, −3.53 and −3.64 eV, respectively. Interestingly, the PIDTT-O and PIDTT-S have the similar LUMO levels, while the LUMO levels of PIDT-O and PIDT-S are also close to each other, because they contain the same IDTT and IDT units in the polymer backbones, respectively. In addition, the HOMO levels of these polymers have the same features. The PIDT-O and PIDT-S show the lower HOMOs than PIDTT-O and PIDTT-S. This is because that the IDTT unit in PIDTT-O and PIDTT-S exists the higher conjugation length than IDT unit in the PIDT-O and PIDT-S, which are in good agreement with the published results [19,20,21,22,23]. It is well-known that the open circuit voltage (*V_oc_*) is related to the energy level difference between the donor’s HOMO and the acceptor’s LUMO [22]. Based on the HOMO energy levels of PIDT-O, PIDTT-O, PIDT-S and PIDTT-S here, it can be predicted that the photovoltaic performance in PSCs would lead to the high *V_oc_* values.

### 2.4. Hole Mobilities

**Figure 3:**
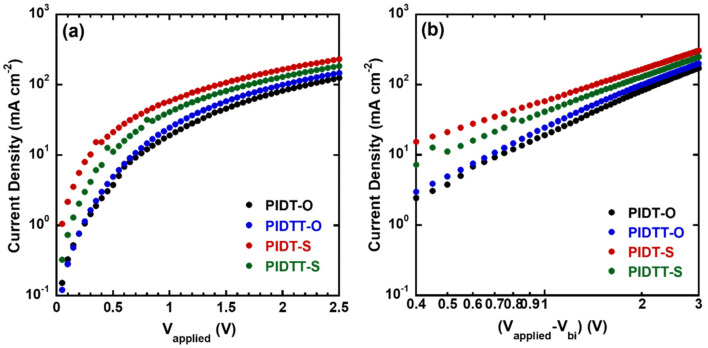
(**a**) The J-V curves of hole-only devices for neat polymers, and (**b**) fitting results to (**a**) from the SCLC model.

To study the charge-transport properties of the PIDT-O, PIDTT-O, PIDT-S and PIDTT-S, the space-charge-limited current (SCLC) method was performed to investigate thoroughly the hole mobility of neat polymers. The hole mobilities (*µ_h_*) from Mott-Gurney equation was measured based on the hole-only devices with the devices structure of ITO/PEDOT: PSS/polymer donor (100 nm)/MoO_3_/Al, where the current density−voltage (*J-V*) curves from hole-only devices were presented in Figure 3 and the related data were summarized in Table 3. As shown in Figure 3, it displays the typical *J-V* curves of the hole-only devices and the corresponding data are summarized in Table 2. It is found that these four polymer donors exhibit the *µ_h_s* of 6.01 × 10^−4^ for PIDT-O, 7.72 × 10^−4^ for PIDTT-O, 1.83 × 10^−3^ for PIDT-S, and 1.29 × 10^−3^ cm^2^ V^−1^ s^−1^ for PIDTT-S, respectively. It is clear that the PIDT-S and PIDTT-S based devices show the relatively higher hole mobilities than PIDT-O and PIDTT-O based counterparts. These higher hole mobility values in PIDT-S and PIDTT-S are possibly resulted from the stronger S-based noncovalent conformational interaction between polymer chains [18,25]. This result implies that the PIDT-S and PIDTT-S based bulk-heterojunction PSCs would support the better hole-transport performance and ultimately lead to the higher photovoltaic properties.

### 2.5. Photovoltaic Properties

**Figure 4:**
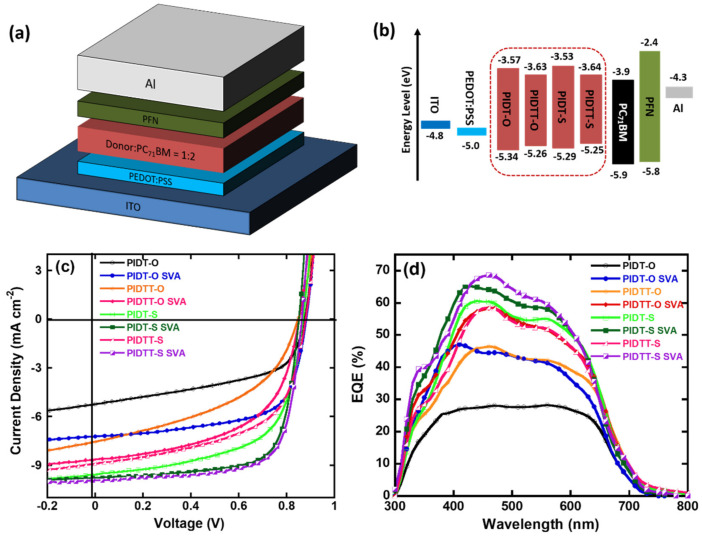
(**a**) The architecture of BHJ devices. (**b**) Energy level diagram of the photovoltaic materials. (**c**) *J-V* curves; and (**d**) the external quantum efficiency spectra of the BHJ photoactive OSCs.

To investigate the photovoltaic performance, the BHJ PSCs with PIDT-O, PIDTT-O, PIDT-S and PIDTT-S as electron donors were explored in detail, whereby the fullerene derivative PC_71_BM was used as the electron acceptor. The relatively conventional devices were assembled with a configuration of ITO/PEDOT: PSS (40 nm)/active layer (80–100 nm)/PFN (5 nm)/Al (100 nm) as presented in Figure 4a. The donor/acceptor (D/A) weight ratio was 1:2 for all the BHJ PSCs. The energy diagrams of the acceptor PC_71_CM was shown in Figure 4b with HOMO and LUMO levels of −5.9, and −33.9 eV, respectively. The difference of LUMOs between the donor and acceptor materials is near above 0.3 eV, which is sufficient for charge separation [26,27]. Through photovoltaic characterization, the J-V curves were recorded in Figure 4c and the corresponding data were summarized in Table 4. It is noted that the devices without any other post-treatment show lower PCEs of 2.41%, 2.89%, 5.09% and 4.43% for PIDT-O, PIDTT-O, PIDT-S, and PIDTT-S, respectively. Based on these devices, all of them give the high *V_oc_s* over 0.85 V ascribing to their lower HOMO energy levels. Compared with the PIDT-O and PIDTT-O based devices, the ones based on PIDT-S and PIDTT-S display the higher PCEs, possibly resulting from their higher hole mobilities than PIDT-O and PIDTT-O. The higher hole mobilities would supply the better hole transport channel and suppress the negative charge carriers recombination in the BHJ systems.

Besides, in order to improve the photovoltaic performance, we used solvent vapor annealing (SVA, TFH) technique to promote the miscibility and molecular orientation in the active layer. As shown in Figure 4c and Table 4, after THF SVA, all of the PIDT-O, PIDTT-O, PIDT-S and PIDTT-S based devices exhibit the higher PCEs of 4.12%, 4.06%, 6.05%, and 6.12%, respectively. In particular, the fill factors (FFs) realize the greater increase from 52.39%, 45.02%, 60.42% and 56.66% to 64.83%, 55.22%, 72.04% and 71.79% for PIDT-O, PIDTT-O, PIDT-S, and PIDTT-S, respectively. We can see that the PIDT-S and PIDTT-S based devices show the better enhancement in FFs exceeding to 71%, which indicate that the nano-scale phase separation would be improved tremendously after THF SVA [28,29,30]. Interestingly, when the SVA was used, there is rare change in *V_oc_s*, whereas the *J_sc_* values realize a little improvement to 7.22, 8.64. 9.76, and 9.92 mA cm^−2^ for PIDT-O, PIDTT-O, PIDT-S and PIDTT-S, respectively.

The external quantum efficiency (EQE) spectra of the conventional and SVA optimized devices for PIDT-O, PIDTT-O, PIDT-S and PIDTT-S as polymer donors were shown in Figure 4d. The devices show the effective photo response in the range from 300 to 700 nm region, which is associated with the UV-vis absorption spectra in the neat films. It is clear that the absorption of donors plays a significant role in the photocurrent spectra. Compared with PIDT-O and PIDTT-O based devices, those of PIDT-S and PIDTT-S based PSCs present the stronger photocurrent responses from 300 to 700 nm, implying the more efficient charge separation and photo harvesting, which are consistent with the higher *J_sc_s* and PCEs.

### 2.6. Atomic Force Microscopy Topographies

**Figure 5:**
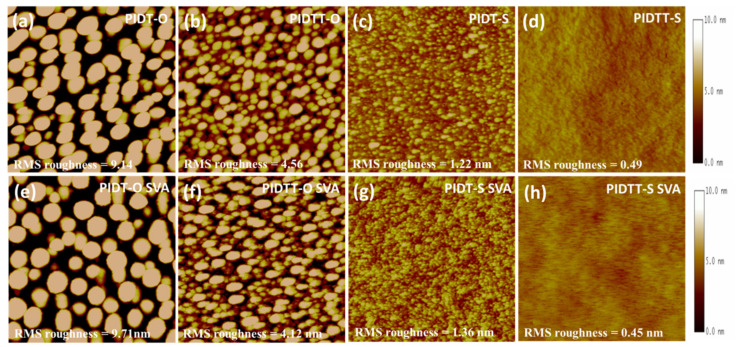
Surface topographic AFM images of polymer: PC_71_BM blend films (**a**) PIDT-O, (**b**) PIDTT-O, (**c**) PIDT-S, (**d**) PIDTT-S without SVA treatment, and (**e**) PIDT-O, (**f**) PIDTT-O, (**g**) PIDT-S, (**h**) PIDTT-S with SVA treatment.

In the view of photovoltaic performance, based on PIDT-O, PIDTT-O, PIDT-S and PIDTT-S, it is obvious that the PIDT-S and PIDTT-S based devices exhibit the higher FF values than those based on PIDT-O and PIDTT-O. One of the reasons for these high FFs is possibly because the PIDT-S and PIDTT-S based blend films would form the better nano-scale phase separation than PIDT-O and PIDTT-O, and realize the optimal bi-continuous D/A phases. Hence, the morphology of the D/A phases is crucial to the performance of photovoltaic devices. Here, the atomic force microscopy (AFM) was employed to investigate the surface topographies of the active layers with the polymers as the donors and PC_71_BM as acceptor [31]. The AFM images of these blend films were shown in Figure 5. It illustrates that the PIDT-O and PIDTT-O based blend films show the very rougher surface with the root mean square (RMS) roughness values of 9.14 and 4.56 nm, respectively. Even though, the RMS roughness also stays very high for PIDT-O and PIDTT-O-based blend films after SVA. Comparably, the PIDT-S and PIDTT-S give the very flat topographies with the 1.22 and 0.45 nm, respectively. Based on the AFM topography characterization, we can see that the PIDT-O and PIDTT-O based active layers would form the vigorous phase separation and lead to lower photovoltaic performance, which is in good agreement with the results from the PSCs characterization.

## 3. Experimental Section

### 3.1. Characterization and Instrumentation

To characterize the polymer structure, the ^1^H NMR spectra were tested by using Bruker (500 MHz) DRX spectrometer (Bruker, Karlsruhe, Germany) with tetramethylsilane (TMS) as the internal reference. Through utilizing the linear polystyrene (PS) as internal standards and tetrahydrofuran (THF, J&K Corp., Beijing, China) as eluent, the number-averaged molecular weight (*M_n_*) and weight-averaged molecular weight (*M_w_*) of the final polymers were measured on Waters gel permeation chromatography (GPC). The thermal gravimetric analysis (TGA) was performed on the TG 209 F3 Tarsus (NETZSCHC, Selb, Germany). Cyclic voltammetry (CV) measurement was employed on a PARSTAT2273 electrochemical workstation electrochemical workstation (Princeton Instruments, Trenton, NJ, United States) in the anhydrous acetonitrile under the nitrogen protection with a tetrabutylammonium hexafluorophosphates (Bu_4_NPF_6_, 0.1 mol L^−1^) solution as electrolyte, accompanied by using a standard three electrodes cell with a Pt wire counter electrode, a platinum (Pt) working electrode, against saturated calomel electrode (SCE) as reference electrode. In the CV measurement, the ferrocene/ferrocenium (*Fc*/*Fc*^+^) was utilized as the internal reference. To get the UV-vis absorption, it is performed on a SHIMADZU UV-2700 spectrophotometer (SHIMADZU, Kyoto, Japan). To obtain the topography images of polymer: [6,6]-phenyl-C71-butyric acid methyl ester (PC_71_BM, purchased from ADS Corp., Cambria, CA, United States) based active layers, we used the atomic force microscopy (AFM) under the tapping-mode on a Veeco Nanoscope V scanning probe microscope to test topographies. The film thickness was measured on a Dektak XT step profiler (Bruker, Billerica, MA, United States).

### 3.2. Device Fabrication

In this work, the indium tin oxide (ITO)-coated glass (the size is 15 mm × 15 mm with the square resistance of 15 Ω) was used as the conductive substrate for fabricating PSCs. Prior to using the ITO substrate, it was treated by UV-ozone process Then, a layer (40 nm) of conductive polymer poly(3,4-ethylenedioxythiophene): poly(4-styrenesulfonate) (PEDOT: PSS) (Clevios 4083) was spin-coated onto ITO within 3000 rpm and then baked at 140 °C for 15 min in air. The active layer was prepared from the mixed solution containing polymers and PC_71_BM with the weight ratio of 1:2 in chlorobenzene, where the spin-coat speed was 1200 rpm during 40 s inside a glove-box with nitrogen. For the photovoltaic devices, the conventional device structure with ITO/PEDOT-PSS/active layer/PFN/Al was utilized, in which the PFN is a alcohol soluble polymer of poly[(9,9-dioctyl-2,7-fluorene)-alt-(9,9-bis (3′-(*N*,*N*-dimethylamino)propyl)-2,7-fluorene)]. Furthermore, a PFN layer (5 nm) was prepared above the active layer by spin-casting a mixed solution (0.2 mg mL^−1^) in methanol solution with a trace of acetic acid. Finally, the cathode (80 nm) was prepared by thermally evaporating the aluminum under vacuum (~10^−6^ torr) with a shadow mask of 0.16 cm^2^. To characterize the photovoltaic performance, the current-voltage (*J*-*V*) curves were tested on a Keithley 2400 multimeter under standard solar illumination (AM 1.5 G, 100 mW cm^−2^). The external quantum efficiency (EQE) were measured by a monochromator under calibrating with a silicon photodiode. The hole mobility of neat polymer films was tested by space-charge-limited current (SCLC) method with the device configuration of ITO/PEDOT-PSS/neat polymers/MoO_3_/Al.

### 3.3. Synthesis of Polymers

The starting monomers of 4,7-Bis(5-bromothiophen-2-yl)-5,6-bis(octyloxy)benzoxadiazole 1 and 4,7-bis(5-bromothiophen- 2-yl)-5,6-bis(octyloxy)benzothiadizole 2 were synthesized according to the our published literatures [22,23], where the indacenodithiophene (IDT, 3) and indacenodithieno [3,2-b] thiophene (IDTT, 4) derivatives were purchased commercially (Solarmer Corp., Beijing, China). The final polymers in this work were prepared by the following general Stille reaction.

#### 3.3.1. General Procedure for Preparing Polymers

4,7-Bis(5-bromothiophen-2-yl)-5,6-bis (octyloxy) benzoxadiazole 1 or 4,7-Bis(5-bromothiophen- 2-yl)-5,6-bis (octyloxy) benzothiadizole 2 (0.3 mmol), and indacenodithiophene (IDT, 3) or indacenodithieno [3,2-b]thiophene (IDTT, 4) derivatives (0.3 mmol), Pd_2_(dba)_3_ (11.0 mg, 0.012 mmol) (J&K Corp., Beijing, China) and tri-o-tolylphopine (18.3 mg, 0.06 mmol) (J&K Corp., Beijing, China)were dissolved in 6 mL xylene under nitrogen. The mixture was then heated to 120 °C and continued to react for 48 h. After 48 h, the solution was cooled to room temperature, and precipitated in methanol, respectively. The crude red polymers were then purified by using Soxhlet extraction in methanol, acetone, hexane, and chloroform, respectively. At last, the chloroform solution, comprising the target polymers, was precipitated in pure methanol, filtered off under vacuum and then dried at 60 °C in the vacuum overnight.

#### 3.3.2. Poly(indacenodithiophene-alt-4,7-di(thiophen-2-yl)-5,c-bis(octyloxy) benzoxadiazole) (PIDT-O)

A red solid as target polymer was achieved with the yield of 510 mg (88.4%). ^1^H NMR (CDCl_3_, 500 MHz, δ): 8.46–8.38 (m, 2H, Ar-H), 7.43–7.37 (m, 2H, Ar-H), 7.31–7.28 (br, 2H, Ar-H), 7.23–7.08 (br, 18H, Ar-H), 4.18 (br, 4H, CH_2_), 2.58–2.57 (m, 8H, CH_2_), 2.07–2.01 (br, 4H, CH_2_), 1.65–1.60 (br, 4H, CH_2_), 1.50–1.30 (br, 48H, CH_2_), 0.88–0.86 (m, 18H, CH_3_). GPC (THF): *M_n_* = 26,300, *M_w_* = 45,000, PDI = 1.71.

#### 3.3.3. Poly(indacenodithieno[3,2-b]thiophene-alt-4,7-di(thiophen-2-yl)-5,6-bis(octyloxy) benzoxadiazole) (PIDTT-O)

A red solid as target polymer was achieved with the yield of 470 mg (76.6%). ^1^H NMR (CDCl_3_, 500 MHz, δ): 8.47–8.40 (m, 2H, Ar-H), 7.53–7.47 (m, 4H, Ar-H), 7.34–7.28 (br, 4H, Ar-H), 7.23–7.05 (br, 14H, Ar-H), 4.20 (br, 4H, CH_2_), 2.58 (br, 8H, CH_2_), 2.04 (br, 4H, CH_2_), 1.64–1.60 (br, 4H, CH_2_), 1.55–1.29 (br, 48H, CH_2_), 0.88–0.85 (m, 18H, CH_3_). GPC (THF): *M_n_* = 43,700, *M_w_* = 91,200, PDI = 2.09.

#### 3.3.4. Poly(indacenodithiophene-alt-4,7-di(thiophen-2-yl)-5,6-bis(octyloxy) benzothiadizole) (PIDT-S)

A red solid as target polymer was achieved with the yield of 450 mg (77.4%). ^1^H NMR (CDCl_3_, 500 MHz, δ): 8.56–8.48 (m, 2H, Ar-H), 7.47–7.37 (m, 2H, Ar-H), 7.30–7.28 (br, 2H, Ar-H), 7.23–7.07 (br, 18H, Ar-H), 4.14 (br, 4H, CH_2_), 2.58–2.57 (m, 8H, CH_2_), 2.04–1.96 (br, 4H, CH_2_), 1.66–1.59 (br, 4H, CH_2_), 1.48–1.29 (br, 48H, CH_2_), 0.87–0.85 (m, 18H, CH_3_). GPC (THF): *M_n_* = 26,800, *M_w_* = 53,400, PDI = 1.99.

#### 3.3.5. Poly(indacenodithieno[3,2-b]thiophene-alt-4,7-di(thiophen-2-yl)-5,6-bis(octyloxy) benzothiadizole) (PIDTT-S)

A red solid as target polymer was achieved with the yield of 452 mg (73.1%). ^1^H NMR (CDCl_3_, 500 MHz, δ): 8.58–8.52 (m, 2H, Ar-H), 7.52–7.49 (m, 4H, Ar-H), 7.34–7.28 (br, 4H, Ar-H), 7.24–7.08 (br, 14H, Ar-H), 4.1 (br, 4H, CH_2_), 2.58 (br, 8H, CH_2_), 2.04–1.98 (br, 4H, CH_2_), 1.64–1.60 (br, 4H, CH_2_), 1.50–1.25 (br, 48H, CH_2_), 0.87–0.85 (m, 18H, CH_3_). GPC (THF): *M_n_* = 49,400, *M_w_* = 120,100, PDI = 2.43.

## 4. Conclusions

In summary, four wide band-gap polymers PIDT-O, PIDTT-O, PIDT-S and PIDTT-S were designed, synthesized and used as donor materials for fullerene-based BHJ PSCs. As a result of the introduction of planar IDT and IDTT units to the polymer main chains, the target polymers showed high hole mobilities, which would increase the intramolecular charge transfer from donor to acceptor phases, and thus, the charge transport in PSCs is enhanced effectively. All polymers exhibited the impressive hole mobility as high as 6.01 × 10^−4^, 7.72 × 10^−4^, 1.83 × 10^−3^ and 1.29 × 10^−3^ cm^2^ V^−1^ s^−1^ for PIDT-O, PIDTT-O, PIDT-S and PIDTT-S, respectively. The sulfur-substituted (octyloxy)benzothiadizole derivatives (PIDT-S and PIDTT-S) presented the higher hole mobilities than oxygen-substituted (octyloxy)benzoxadiazole derivatives (PIDT-O and PIDTT-O). Through the photovoltaic characterization, the PIDT-S and PIDTT-S based PSCs gave the higher PCEs of 5.09% and 4.43% than PIDT-O and PIDTT-O, due to their higher hole mobilities. Interestingly, the SVA technique can improve the photovoltaic performance dramatically with the PCEs reaching 4.12%, 4.06%, 6.05% and 6.12% for PIDT-O, PIDTT-O, PIDT-S and PIDTT-S, respectively. This obvious enhancement on PCEs were mainly resulted from the distinct improvement on FF values, which suggested that the SVA is an effective methodology for improving photovoltaic properties. These results indicated that these wide band-gap polymers could be promising candidates for the fabrication of high-performance BHJ PSCs.

## Figures and Tables

**Table 1:** Molecular weights and thermal properties for the polymers.

Polymers	*M_n_*	*M_w_*	PDI	*T_d_* (°C)
PIDT-O	26,300	45,000	1.71	337
PIDTT-O	43,700	91,200	2.09	343
PIDT-S	26,800	53,400	1.99	335
PIDTT-S	49,400	120,100	2.34	362

**Table 2:** Optical and electrochemical properties of PIDT-O, PIDTT-O, PIDT-S and PIDTT-S.

Polymers	*λ_abs_*, Solution (nm)	*λ_abs_*, Film (nm)	*λ_abs_*, Onset (nm)	*E_g_*, Opt (eV)	*E_ox_* (eV)	*E_red_* (eV)	*E_HOMO_* (eV)	*E_LUMO_* (eV)
PIDT-O	439,573	426,570,600	660	1.88	0.92	−0.85	−5.34	−3.57
PIDTT-O	453,574	438,564,598	661	1.87	0.84	−0.79	−5.26	−3.63
PIDT-S	447,556	436,558	655	1.89	0.87	−0.89	−5.29	−3.53
PIDTT-S	453,553	448,556	650	1.91	0.85	−0.78	−5.25	−3.64

**Table 3:** Summary of the hole mobilities of the neat polymers.

Polymers	Thickness (nm)	Hole Mobility (cm^2^ V^−1^ s^−1^)
PIDT-O	100	6.01 × 10^−4^
PIDTT-O	100	7.72 × 10^−4^
PIDT-S	100	1.83 × 10^−3^
PIDTT-S	100	1.29 × 10^−3^

**Table 4:** Photovoltaic performances of PSCs based on PIDT-O, PIDTT-O, PIDT-S and PIDTT-S under the illumination of AM 1.5 G, 100 mW cm^−2^.

Polymers	*V_oc_* (V)	*J_sc_* (mA cm^−2^)	FF (%)	PCE (%)
PIDT-O	0.88	5.22	52.39	2.41
PIDT-O SVA ^a^	0.88	7.22	64.83	4.12
PIDTT-O	0.85	7.56	45.02	2.89
PIDTT-O SVA	0.85	8.64	55.22	4.06
PIDT-S	0.88	9.58	60.42	5.09
PIDT-S SVA	0.86	9.76	72.04	6.05
PIDTT-S	0.88	8.89	56.66	4.43
PIDTT-S SVA	0.86	9.92	71.79	6.12

^a^: SVA means THF assisted solvent vapor annealing.
